# Automated breast volume scanner: an initial experience

**DOI:** 10.1186/bcr2947

**Published:** 2011-11-04

**Authors:** EK Hughes, L Nassar, A Lim, N Barrett, S Comitis, D Cunningham, S Flais, A Gupta, G Ralleigh, V Stewart, W Svensson, R Williamson, N Zaman, K Satchithananda

**Affiliations:** 1 Charing Cross Hospital, Imperial College Healthcare NHS Trust, London, UK

## Introduction

The automated breast volume scanner (ABVS) is the first of its kind and utilises a large, 17 cm × 15 cm high-frequency ultrasound probe which sweeps across the whole breast generating images that can be reformatted into multiple planes and a 3D volume. ABVS will change breast ultrasound practice by: introducing operator standardisation, reproducibility and repeatability of measurement and interpretation; changing who acquires the volume set and how breast ultrasounds are reported; and allowing accurate comparison of previous and current examinations for screening and assessing treatment change.

## Methods

Patients presented to the symptomatic clinic for conventional 2D ultrasound assessment with a variety of conditions. An additional ABVS was performed.

## Results

Cases were classified into: benign - for example, cysts, fibroadenomas, diabetic mastopathy; and malignant.

## Conclusion

We present a review of our initial experience and highlight its advantages over conventional ultrasound, which include: improved mapping of lesions enabling more accurate future assessment and follow-up, and improved assessment of distortion over conventional 2D ultrasound. Further research is required to explore other potential benefits.

